# Unsupervised Learning for Product Use Activity Recognition: An Exploratory Study of a “Chatty Device”

**DOI:** 10.3390/s21154991

**Published:** 2021-07-22

**Authors:** Mike Lakoju, Nemitari Ajienka, M. Ahmadieh Khanesar, Pete Burnap, David T. Branson

**Affiliations:** 1 Cardiff School of Technologies, Cardiff Metropolitan University, Western Avenue, Cardiff CF5 2YB, UK; 2 Department of Computer Science, Nottingham Trent University, Nottingham NG11 8NS, UK; nemitari.ajienka@ntu.ac.uk; 3 Faculty of Engineering, The University of Nottingham, University Park, Nottingham NG7 2RD, UK; ezzma5@exmail.nottingham.ac.uk (M.A.K.); ezzdtb@exmail.nottingham.ac.uk (D.T.B.); 4 School of Computer Science & Informatics, Cardiff University, Wales CF10 3AT, UK; burnapp@cardiff.ac.uk

**Keywords:** Chatty Factories, Industry 4.0, machine learning, IoT, product use activity, sensors, clustering

## Abstract

To create products that are better fit for purpose, manufacturers require new methods for gaining insights into product experience in the wild at scale. “Chatty Factories” is a concept that explores the transformative potential of placing IoT-enabled data-driven systems at the core of design and manufacturing processes, aligned to the Industry 4.0 paradigm. In this paper, we propose a model that enables new forms of agile engineering product development via “chatty” products. Products relay their “experiences” from the consumer world back to designers and product engineers through the mediation provided by embedded sensors, IoT, and data-driven design tools. Our model aims to identify product “experiences” to support the insights into product use. To this end, we create an experiment to: (i) collect sensor data at 100 Hz sampling rate from a “Chatty device” (device with sensors) for six common everyday activities that drive produce experience: standing, walking, sitting, dropping and picking up of the device, placing the device stationary on a side table, and a vibrating surface; (ii) pre-process and manually label the product use activity data; (iii) compare a total of four Unsupervised Machine Learning models (three classic and the fuzzy C-means algorithm) for product use activity recognition for each unique sensor; and (iv) present and discuss our findings. The empirical results demonstrate the feasibility of applying unsupervised machine learning algorithms for clustering product use activity. The highest obtained F-measure is 0.87, and MCC of 0.84, when the Fuzzy C-means algorithm is applied for clustering, outperforming the other three algorithms applied.

## 1. Introduction

As “Industry 4.0” draws significant attention, a large number of organizations are seeking novel methods and techniques of acquiring and processing huge amounts of sensor data. This is considered the birth of a new industrial revolution [1,2]. The first industrial Revolution came as a result of the mechanization of production, which affected product volume dimension. The second Revolution altered the industry by leveraging on the advent of electricity and mass production. The third Revolution was characterized by the adoption of Information Technology (IT) and electronics to process automation [3,4]. More recent discussions are suggestive of the birth of the fourth Industrial Revolution, with the emergence of “Industry 4.0” and “Smart Factory”. By implication, product design and manufacturing within the Industry 4.0 paradigm is now more data-driven.

This most recent revolution in technology offers enormous benefits to product design teams [5,6], such as the capability to leverage sensors to acquire data directly from products. Understanding customers’ needs, satisfaction, experience, etc., form a critical aspect of creating products that satisfy segments of customers and increasing profitability. Manual processes used to collect customer’s feedback can now be automated by leveraging sensors. This, in turn, enables the creation and automation of data-driven processes/strategies for acquiring meaningful product use information that could influence the creation of new products or the enhancements of existing ones.

Advancements in sensor technology have exponentially increased the number of Internet-connected products being produced in factories all around the world today. This, in turn, creates the possibility of collecting massive amounts of product use data that can now be leveraged by product design teams [5,6,7]. This is akin to Opresnik et al.’s [8] opinion that gaining an understanding of product use activities is a data-driven process of information, which is a feedback loop of collecting, storing, and then analyzing data from the product-users with a primary goal to discover and identify usage patterns that inform design suggestions. Therefore, design teams are equipped to tailor products to customers’ needs and wants.

Exploring product-use data strategically gives design engineers a competitive advantage by enabling them to uncover patterns, innovative insights, and knowledge through data-driven design [9]. Conventional product design involves a technical decision process that is characterised by the intrinsic performance of the product [10]. Typically, hypothetical market studies are acquired as a collection of design specifications or based on experiential knowledge, which is subjective and consequently deviates from true customer satisfaction [11]. Interviews, user experiments, focus groups, etc., were a few of the typical methods used in acquiring customer requirements. However, this was limiting because only the customers’ actions and extrinsic behaviors could be captured. Ultimately, this failed to provide latent customer needs and intrinsic requirements for product performances [12].

The data-driven analysis approach towards product design is grounded on the premise that, when manufacturers know how customers are using the products, they can tailor their products much better to actual needs. This enables design decisions to be based on facts and not assumptions [9]

**Figure 1:**
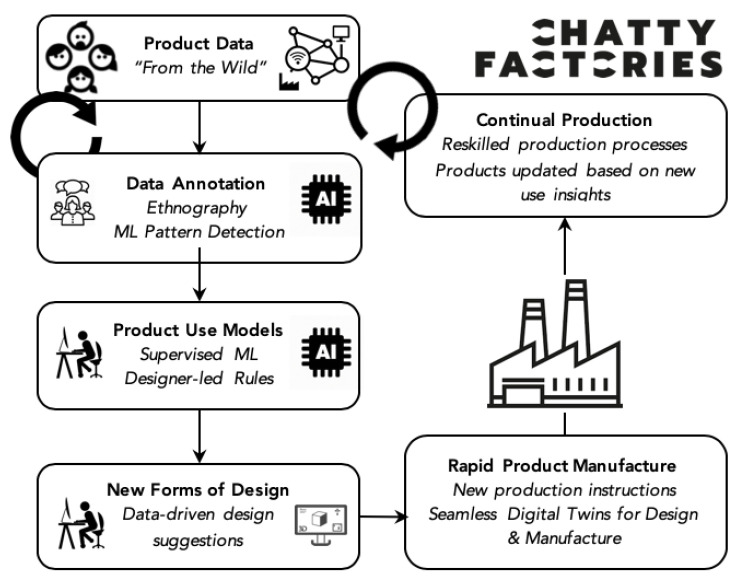
The Chatty Factories vision.

The Chatty Factories concept focuses on the opportunity to collect data from IoT-enabled sensors embedded in products (Chatty devices) during real-time use by consumers, explores how that data might be immediately transferred into usable information to inform design, and considers what characteristics of the manufacturing environment might optimise the response to such data. Figure 1 illustrates the Chatty Factories concept. A detailed background can be found in Reference [13]. In summary, the concept offers the potential to accelerate product refinement through a radical product interruption of ‘consumer sovereignty’ based around surveys and market research, to ‘use sovereignty’ and an embedded understanding of consumer behavior—making products that are fit for purpose based on how they are used. Achieving continuous product refinement in response to real-time data on product use requires digital tools that can interact with physical and virtual life. The “Data Annotation” block in Figure 1 is one of the key phases of the proposed data driven model which requires the use of machine learning models to identify anomalies, patterns, and points of interest in the data. This is the area we address in this paper.

Sensor data streaming from the wild can hold key information about how the product is used by consumers across different locations and cultures. Gathering data from use in the wild can limit the need for current product research studies, such as post-hoc lab studies and user experience surveys. Furthermore, in-situ research methods can capture the intricate and messy relationships between people and products, which may be difficult to assess through lab-studies [14].

Due to the volume of real-time data created by the sensors and the fact that products will be used by a large scale of customers, it becomes necessary to employ the use of unsupervised machine learning models to identify anomalies, patterns, and points of interests at scale. In general, labeling real time activity data is very difficult. The main reason is that, within a single experiment, there may be various types of activities which makes labeling data very difficult, if not impossible. To this end, this paper compares a number of unsupervised machine learning models. Their successful implementation provides evidence to use these approaches for future unlabeled data used for dealing with product activity analysis in a Chatty Factories framework.

Today, the competitive business environment is putting more pressure on manufacturers to constantly improve/manufacture products that satisfy customer demands, usually through changes in design and functionality, relying primarily on continuous analysis of large amounts of data. Product data can be obtained from varying sources; however, technology advancements in sensor technology has inspired many intelligent products—“product embedded information devices (PEID)” [15]. Furthermore, The Internet of Things (IoT), being an ecosystem of such inter-connected devices, makes data transfer even more possible, thus enabling more efficient product lifecycle data. Products retrofitted with a wide array of sensors improve the product’s capability to store, compute, and communicate data (chatty devices), for example, smartphones, tablets, smart wearables, water monitor, etc. [16,17,18].

Leveraging product use data/PEID within a well thought out data-driven methodology becomes a more precise customer feedback method [15] than the manual processes of collecting customer feedback previously utilised in product design [19]. It, therefore, becomes imperative to build on work done around activity recognition that sought to understand people’s daily lives. In this paper, we seek to understand actual product experiences, using activity leveraging unsupervised learning methods.

Activity recognition is a well explored research topic. Researchers have typically focused on Human activity recognition (HAR) because it provides insight into understanding the daily lives of people inferred from analysis carried out from raw sensor inputs [20]. Scholars have successfully investigated Human Activity Recognition in areas, such as home behavior analysis [21], gesture recognition [22], gait analysis [23], video surveillance [24], etc.

Real-time feedback acquired from the actual product-use through sensors embedded in the products would be extremely valuable for product designers to understand how the product is being used. Human behavior identification/classification by interaction with the product can play a crucial part in understanding how the product is being used. But so can producing experiences beyond human activity. For example, a product may roll off an uneven surface, or be transported in a rough manner—neither are always under the direct control of the human. We argue that this additional information about how products “experience the world” will potentially offer new insights for product design teams.

This study focuses on product activity for the purposes of understanding experiences in the wild, and informing future versions of its design and manufacture. It seeks to identify actual product use activity by creating experiments using a “Chatty Device” with inbuilt sensors, such as Accelerometer, Orientation sensor, Magnetic field, and Angular Velocity. Since data used in this study suffers from high dimension, a dimension reduction stage is required. Hence, unsupervised learning approaches because of their power to reduce the data dimension are the preferred approach in this paper [25]. A sampling rate of 100 Hz was used to collect data around common product experiences, such as vibrations, being stationary, drops, and pickups—alongside human activities, while using products, including walking, standing, and sitting. A total of about 148,778 data points were collected.

To our knowledge, general research in activity classification has focused on areas, such as human activity classification, health-related studies, smart homes, etc., while we explore and investigate product use activity classification in the wild for the first time. We approach the problem as an unsupervised learning problem, even though we have created label data. Our rationale is to provide verifiable evidence that allows us to map the actual product use activity to the identified cluster detected by the algorithm.

In this study, we utilize securely and ethically collected product-use big data to investigate how unsupervised learning algorithms can be used to understand at scale, product use in the wild. This is key to the Chatty Factories concept, which is an innovative industry 4.0 model. In this framework, human behavior identification is crucial as it will provide valuable information about how and where the products is used for further analysis by the design team. Product behavior associated with different human activity in terms of vibrations and orientations met by the product provide valuable insight for the design team for possible reinforcement of the product to deal with the situations met by the product as a result of different product use activities. We log sensor data, pre-process it and label the data according to the product use activity carried out. The activity labels created are only for validating the clusters mapped to a specific activity. This helps to evaluate the effectiveness of the clustering method used, thus enabling the authors to justify the approach of using Unsupervised Machine learning to identify unknown product use activities. The standpoint of the authors is that Unsupervised Machine Learning methods can be used effectively to cluster similar product use activities, even if initially unknown. An unknown product use activity “X” can be discovered, and, after an ethnographic intervention, a label can be subsequently assigned to “X”, for example, “A drop”.

The following are the main contributions of this study:

- the first approach to detect discrete product use activities in the wild (vibrations, being stationary, drops, and pickups), enhancing previous approaches that focus only on human activity (walking, standing, and sitting). Product behavior within each product use activity can then be studied by the design team for further investigation and possible modifications for the product;
- the use of the Fuzzy C-means algorithm to effectively and efficiently detect product use activities. The behavior of product associated with product activities would then be easy to study as, instead of dealing with large amount of data within each cluster, the center of cluster can be studied; and
- a novel (publicly available) curated and manually verified dataset consisting of actual product use activity allowing researchers to conduct further studies in this domain and compare machine learning results (Dataset is available at: https://doi.org/10.6084/m9.figshare.11475252.v1, accessed on 3 December 2020) curated and manually verified dataset consisting of actual product use activity (for further comparative studies) allowing researchers to conduct further studies in this domain and compare machine learning results).

The rest of the paper is structured as follows: Section 2 provides an overview of related studies on activity detection. Section 3 discusses the materials used in the study, such as the sensors, dataset, and product use activities. Section 4 is the Methodology section, which describes the empirical approach used to carry out the product use activity clustering using unsupervised machine learning methods, including the data pre-processing and independent feature selection process. Furthermore, Section 4.4 summarizes the results and discusses the findings and provides further empirical insights. Section 5 describes the unsupervised machine learning results, while Section 6 concludes the paper.

## 2. Related Work

This section provides an overview of related research on the application of machine learning techniques for activity detection.

Dealing with product-use activity classification/identification at scale requires the application of machine learning to identify those patterns and anomalies. There are many and varied studies on “human activity classification”; however, the context of primarily focusing on “product-use activity classification” is limited. This has prompted a key objective of this study. It is believed that detecting activities, like picking up the device, dropping the device, or exposing the device to a vibrating environment, etc., can provide useful product design information [8]. For instance, Ankita et al. [26] focused primarily on improving human activity recognition by combining convolutional layers, long short-term memory (LSTM), and the deep learning neural network to extract key features in an automated way and then categorize them with some model attributes. Essentially, their study did not focus on product activity recognition; they tested their model on a publicly available dataset of UCI-HAR for Samsung Galaxy S2, which captures various human activities, such as walking, walking upstairs and downstairs, sitting, standing, and laying. They did not look at activities that could impact a product, like vibrations within the environment. Russell et al. [27] also primarily focused on the context of human activity recognition. They set out to create and validate a field-based data collection and assessment method to aid human activity recognition in the mountains with terrain and fatigue variations. An output from their study was an unsupervised labeled dataset of various long-term field-based activities, such as obstacle climb, stand, run, walk, and lay. Furthermore, they were keen on the human context rather than product classification. As such, they were evaluating Fatigue levels by modulating between rested to physical exhaustion.

In this section, we review literature, focusing on activities, such as “vibration”, “drop”, and “pickup”, and others, like “standing”, “walking”, “sitting”, and “being stationary”, in order to determine whether existing research on activity detection has achieved the detection of these activities particularly within the context of application for product-activity classification. Table 1 summarizes some of the gaps in existing literature.

While still focusing on human activity recognition, Jun and Choi [28], focused on a slightly different area. They argue that lots of studies have been done recognizing Adult human activities; however, HAR would be beneficial for the safety and wellness of newborns or infants because they have poor verbal communication. To this end, they focus their research on newborn and infant activity recognition by analyzing accelerometer data by attaching sensors to the body. They classify four types of activities: sleeping, moving in normal condition, moving in agony, and movement by an external force. Furthermore, they took an unsupervised learning approach torwards recognizing activies leveraging an end-to-end deep model using autoencoder and k-means clustering. Similarly, their study did not take into consideration product activity recognition.

Gao et al. [29] carried out a study on human activity recognition based on Stacking Demonising Autoencoder (SDAE) and LightGBM (LGB). Their approach uses the SDAE to remove noise from the raw sensor data to extract valid characteristics of the data with unsupervised learning. At the same time, the LGB was applied to distinguish the inherent feature dependencies among categories for accurate human activity recognition. They test their algorithm on activities, such as walking, going up and down an Elevator and Escalator, standing, sitting, lying, etc. The study did not consider product-use recognition nor evaluate activities, like vibrations from the environment, that could affect a product.

Wyatt, Philipose, and Choudhury [30] considered unsupervised learning methods in their study positing activities can be differentiated or identified by the objects used. Presented with the names of these objects, they created models of activities from the web, and proposed that the models can distinguish twenty-six (26) activities. However, they did not consider activities, like vibrations, drop and picking up, or stationary position. Huynh [31] proposed an unsupervised learning model that deals with multiple time scales, by leveraging multiple eigenspaces. In their study, activities, such as walking, standing, and sitting, were recognized with four inertial sensors. Li and Dustdar [32] conducted experiments with the anticipation of adopting unsupervised learning methods in activity recognition. They put forward a hybrid process that fuses activity classification with subspace clustering in a bid to handle the curse of dimensionality, but their research did not look into specific activities, like drop and pickup, vibrations, etc., and Vandewiele and Motamed [33] looked into a smart home environment with a network of sensors. They suggest that an unsupervised learning approach would be useful for activity recognition in such an environment; however, they did not consider activities, such as drop and pickup, vibrations, walking, stand-in, sitting, or stationary surface. They also did not focus on the context of application to product-use activity identification.

Trabelsi [34] put forward an unsupervised learning method for human activity recognition by using three accelerometers which were attached to the right thigh, left ankle, and chest. They used the Hidden Markov Model Regression and proposed that their model substantially outperformed other unsupervised learning methods and was closely comparable to supervised learning methods. However, for the application of the algorithms, it is assumed that the number of activities is known. They also did not consider activities, like vibrations or drop and picking up. Furthermore, a number of scholars have focused on human activity recognition by leveraging a combination of sensors which are embedded in cameras [35], mobile devices, wearable computers, etc. Accelerometers are one of the most frequently used sensors for activity recognition because its functionality helps in measuring activities performed by users [36]. Some studies employ the use of multiple accelerometers attached to different locations of the human body. For example, Reference [37] ran experiments that used two or three uniaxial accelerometers to differentiate various activities, which include people lying down, standing, sitting, descending and ascending stairs, and also cycling. They, however, fail to consider activities, like vibration or drop and pickup. Arminian [38] investigated the possibility of using two accelerometers, one attached to the rear of the thigh and the other on the chest. Foerster and Fahrenberg [39] placed three uniaxial accelerometers on the sternum, and two uniaxial accelerometers on the right and left thighs to identify four basic activities (moving, standing, sitting, and lying down).

According to Reference [40], human activity detection can be done using static platforms or wearable platforms. Two major static platforms to detect human motions are marker-based motion capture system [41] and force plate system [42]. The combination of these two static platforms may also be used for human activity detection purposes. Other than static platforms, wearable platforms can be used to detect human motion. Examples of wearable platforms are accelerometers [43], gyroscopes, magnetometers, electro/flexible goniometers [44], and sensing fabrics [45]. The combination of these sensors may also be used for activity detection purposes [40]. In this study, the combination of accelerometers, gyroscopes, and magnetometers is used for product activity detection. The fact that all of these sensors are available under iPhone makes it a cheap product activity detection method.

Previous research typically employed machine learning methods to investigate activity recognition; however, Table 1 highlights some gaps based on some specific activities that could potentially facilitate product design. This study focuses on activity recognition of product use. It presents the application of unsupervised machine learning models on a novel real-time product use activity dataset derived from sensors embedded in a “chatty” product. This will underpin an understanding of how the product is used at scale and could inform product design modification in a way hitherto not possible.

**Table 1:** Summary of activity recognition on vibration, drop and pickup, standing, sitting, walking, and stationary position.

Paper	Activities						Application in Product-Use Activity Identification
	**Drop** & **Pickup**	**Vibrations**	**Walking**	**Standing**	**Sitting**	**Stationary Position**	
[23,26,29,31,34,37,38,39,46,47,48,49,50,51,52,53,54,55,56,57,58,59,60,61,62,63,64,65,66,67,68,69,70,71,72]	0	0	X	X	X	0	0
[27,73,74,75,76,77,78]	0	0	X	X	0	0	0
[23,79,80,81,82,83,84,85,86]	0	0	X	0	0	0	0
[33,87,88,89]	0	0	0	0	0	0	0
[90]	0	0	0	0	X	0	0
[35,91]	0	0	X	0	X	0	0
[30,92]	0	0	0	X	0	0	0
This Paper	X	X	X	X	X	X	X

## 3. Materials

**Figure 2:**
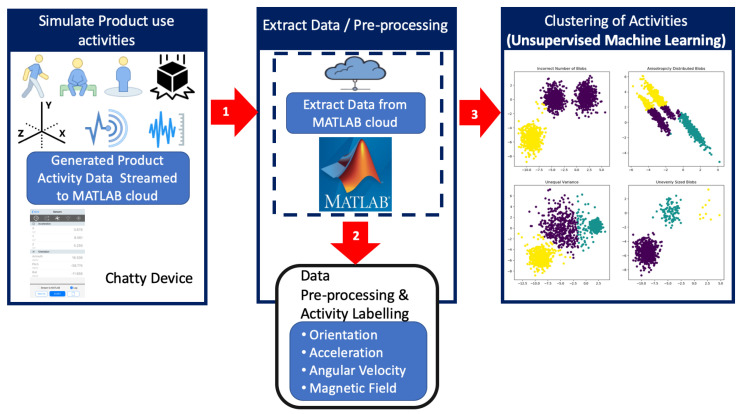
Flow of work.

For this study, a dataset was created by conducting expected product use activities, such as placing the device on a vibrating surface, dropping and picking up of the device, walking with the device, sitting with the device, placing the device stationary on a side table, and standing with the device. A 6th generation Apple iPad was used as the “Chatty device” for this study because it has all the inbuilt sensors that enable the research team to measure and detect these activities. Modern mobile iOS and Android devices are equipped with a rich variety of sensors, thus presenting an ideal medium to collect data for research. Apple devices typically offer a number of functionalities that leverage the inbuilt sensors in the devices. These include proximity sensors, accelerometers, ambient light sensors, gyroscope, and more [93], which, for example, can enable a user shake the device to undo an action or dim the screen when the device is held at face level, etc. Figure 2 illustrates the flow of the experiment.

The data was collected from the following four in-built sensors.

- Acceleration: 3-axes acceleration data,
- Orientation: Azimuth, Pitch, and Roll,
- Angular Velocity: 3-axes gyroscopes for rotational motion,
- Magnetic Field: 3-axes magnetic field.

Acceleration on 3-axes: This sensor is used to measure the linear acceleration of the device. The sensor has the capability to function in two modes ±2 g and ±8 g. Both modes can sample data at 400 Mhz or at 100 Mhz. Apple uses a default of ±2 g mode (100 Mhz) [94].

Azimuth, roll, pitch (Orientation): Another sensor pre-built into the device is a vibrational gyroscope. It functions by utilizing a plate called a “proof mass”. When a signal is applied to capacitor plates, it oscillates. When an individual displaces the device in a rotational manner, the proof mass is displaced in the X, Y, Z directions an ASIC processor measures the capacitance change of the plates [94].

Magnetic Field on 3-axes: This sensor can measure the strength of the magnetic field surrounding the device. Hence, the device is equipped with the ability to determine its “heading” in line with the geomagnetic North Pole, thus acting as a digital compass [94].

Angular Velocity on 3-axes: The angular velocity also known as rotational velocity, can be explained as the rate of velocity that an object or a particle rotates around a defined point at a given time.

## 4. Methodology

In this section, we describe the methodology adopted for conducting the experiments/study.

### 4.1. Data Acquisition

The MATLAB Mobile application was used to stream data from the in-built sensors of the iPad (Chatty-device). MATLAB is a purposed built scientific environment for running experiments. It is created for engineers and scientists with a robust set of Apps and toolboxes prepackaged and rigorously tested for a broad range of scientific and engineering applications [95]. The MATLAB mobile app application is convenient and freely available for individuals with an academic email. Hence, the research team decided to use the MATLAB mobile app, which runs on both Android and iOS devices and is user friendly. Some open-source applications can also collect data from mobile devices; however, the majority of them are platform-dependent and, most times, not free. The product use data was streamed into a MathWorks Cloud drive account at a sampling rate of 100 Hz per activity. The research team collected about four (4) minutes of sensor data for every activity through a total of a total of 148,778 data points. The following steps were taken:

1. The MATLAB mobile app was installed and configured on the Chatty device through the Apple App store.
2. A registered user account was used, and the device was connected to MathWorks Cloud account.
3. Using the MATLAB Mobile interface, the sensors were turned “on”.
4. A sampling rate of 100Hz was selected, and the log button was used to initiate recording of the sensor data readings.
5. After the expected duration, the logged results were saved to the Cloud and given file names which corresponded to the activities carried out.

**Figure 3:**
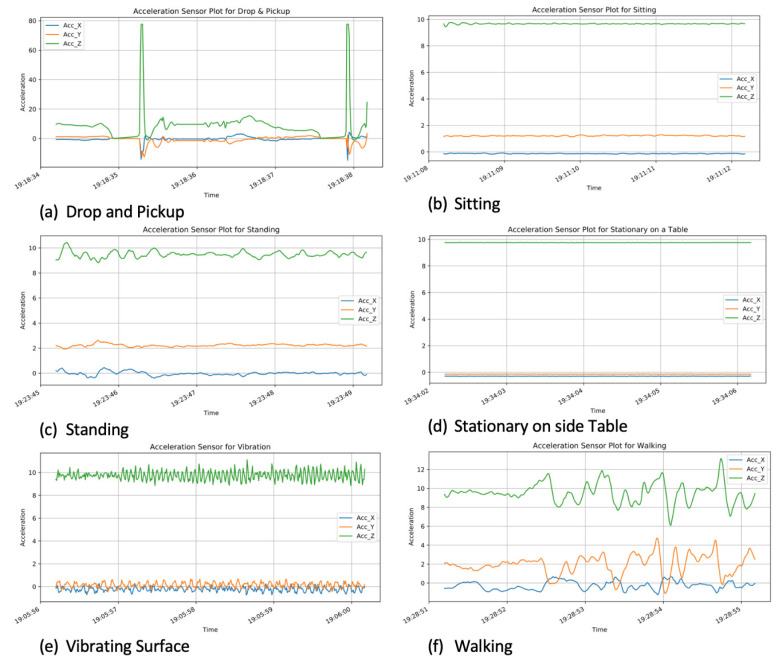
Acceleration signals for the six activities.

Two subjects (an adult female and male) held the device while carrying out the product use activities—dropping and picking up, standing, walking, and sitting. Product use data was also collected while the device was placed on a vibrating and non-vibrating stationary surface. Figure 3 highlights the acceleration plots for the three axes (X, Y, Z) for each of the six activities. Walking is a more energetic activity so when compared with the others, its X, Y, Z values vary significantly. Standing, sitting, device on vibrating surface, and stationary on a side table are more dormant activities; thus, their X, Y, and Z values are almost constant. The drop and pickup activity plots indicate sudden spikes in the signals which come as a result of the sudden drop events that occurred. One interesting thing to observe is that, for walking, standing, sitting, vibrating surface, and stationary on a side table, the “Z” values have the larges accelerations. This is because the force of gravity affects the entire acceleration in the direction of the center of Earth.

**Figure 4:**
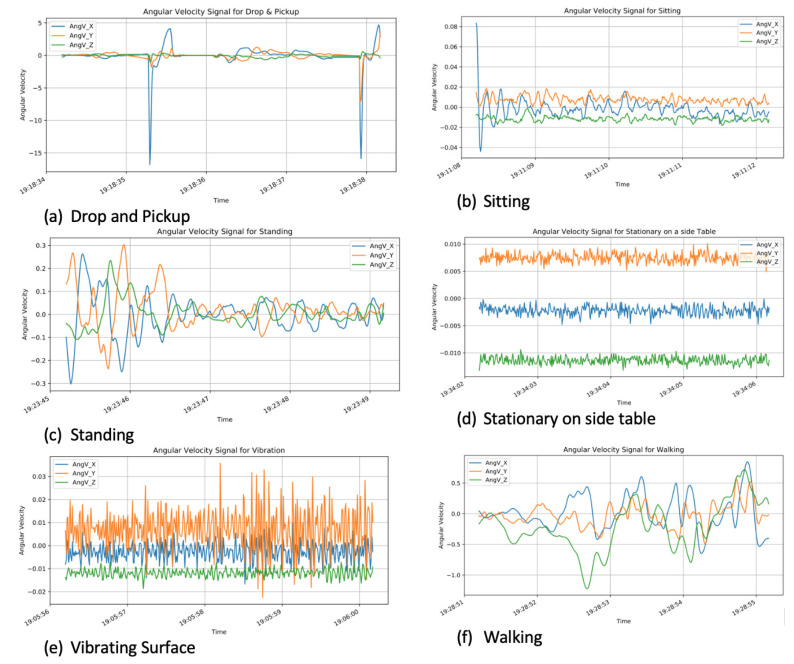
Angular velocity signals for the six activities.

Figure 4 highlights the angular velocity for the three axes (X, Y, and Z) for each of the six activities. Large spikes can also be observed on the drop and pickup activity plot, which indicates the sudden drop events that occurred. For walking and drop and pickup activities, the X, Y, and Z values significantly change. For standing, sitting, stationary on a side table and vibrating surface, the X, Y, and Z values are much smaller (<0.3 rad/s).

### 4.2. Data Pre-Processing and Feature Selection

All the logged product use sensor data was stored in the MathWorks cloud account with a “.mat” file extension. The files were downloaded and imported into MATLAB desktop application. MATLAB allows for variables to be created and used to house data. Hence, a variable was created for each recorded sensor activity. The variable contained the time-stamp and X, Y, Z axis data for each of the sensors. A MATLAB timetable file for each of the sensors was exported to a CSV file and labeled according to each product use activity carried out.

To create the Labels for each activity and assign them to their respective data points, the research team had to preprocess each data table independently before combining them. The MATLAB sensor log file that was downloaded from cloud drive for each activity held the sensor reading for the Accelerometer, Magnetic-field, Orientation, and Angular Velocity sensors. Each sensor Table had timestamp, X, Y, and Z data readings. All sensor tables where then concatenated by columns and then a new column with header “Activity” was created and assigned a value corresponding to the name of the activity carried out. This was done iteratively for each activity distinctively with their labels assigned accordingly. Finally, the research team merged all tables into one master file.

A master data CSV file was created using Microsoft Excel. It contains the combined set of sensor readings of the experiments which were arranged according to the sensors following the sequence of the activities carried out. The timestamp variables were treated as data-time variables on excel and stored accordingly. The CSV file was imported into WEKA for pre-processing and feature selection. WEKA is an open-source data analysis software [96,97] (The tool is available at: http://www.cs.waikato.ac.nz/ml/weka/index.html, accessed on 10 December 2020.) developed by the University of Waikato and has been used with its default setting [96,97,98,99,100].

A total of sixteen (16) independent variables were collected including the following: Acceleration X, Acceleration Y, Acceleration Z, Timestamp-Acceleration, Angular Velocity X, Angular Velocity Y, Angular Velocity Z, Timestamp-Angular Velocity, Magnetic Field X, Magnetic Field Y, Magnetic Field Z, Timestamp-Magnetic Field, Orientation X, Orientation Y, Orientation Z, and Timestamp-Orientation.

Each sensor is unique and, thus, may have a slightly different sampling rate. To this end, we collected data for each of the four sensors and also recorded their respective timestamps to each data-point.

As part of the data preparation exercise, an activity column was created to capture each specific activity. It contained the labeled activities carried out in the experiment, which also represents the dependent or response variable for the experiment. These are the class variables (product use activities) that we seek to detect with the unsupervised learning algorithms

The response variables or classes are six-fold and are as follows:

- dropping and picking up,
- vibrating,
- non-vibrating stationary surface,
- standing,
- walking, and
- sitting.

In prior research [101], the CfsSubsetEval feature evaluator in WEKA has been identified as the evaluator with the best performance for feature selection using its search methods (BestFirst, Ranker search, and Forward selection). In order to remove noisy features from the experiment or features with a weak predictive power, the *BestFirst* feature selection method of the CfsSubsetEval evaluator in WEKA was used to identify a subset of features that have a high predictive ability in relation to the dependent or response variable (product use Activity). In other words, it identifies a subset of features with a high correlation with the classes but low intercorrelation. This feature selection algorithm searches the space of attribute subsets by greedy hill climbing augmented with a backtracking facility.

In addition to the BestFirst technique, a 10-fold cross validation technique was used to identify the features that maintain a high predictive power in all 10-folds. This technique involves randomly dividing the dataset into *k* groups or folds of approximately equal size. The first fold is kept for testing and the model is trained on k−1 folds.

As a result of the feature selection process, the eight (8) features or independent attributes with the highest predictive power and used in the experiment are as follows: Acceleration Y, Angular Velocity X, Angular Velocity Y, Angular Velocity Z, Magnetic Field X, Orientation X, Orientation Y, and Orientation Z.

### 4.3. Finding an Optimal Number of Clusters

This study is aimed at identifying suitable machine learning models for the identification of product use activity in order to relay product usage insights back to product designers, engineers, and manufacturers. This will aid the enhancement of products used in the wild, as well as user satisfaction.

While it is known in advance what activities are in the collected dataset for this study (labeled dataset which should naturally constitute a classification or supervised machine learning problem) having collected the dataset in a controlled environment (as described in Section 3), products are generally used in the wild and the distinct usage activities will not be predetermined. Based on this premise, we want to identify suitable (unsupervised) clustering techniques that can identify product use activities sharing similar attributes and evaluate the performance of these clustering techniques using external performance functions based on the labels (classes) in our dataset. The labels in our dataset are, therefore, solely used to evaluate the accuracy of the clustering algorithms applied in this study, to provide an estimate of how well they will perform on product use datasets gathered from the wild. As we are resolving a clustering problem, it is imperative to identify an optimal number of clusters to use in our experiments, given that the number of product use activities will be unknown in the wild.

Among different methods to find the optimal number of clusters, the elbow method is selected [102]. In this algorithm, the center of clusters is estimated using the K-means clustering algorithm (to perform the clustering) and then the *Within-Cluster-Sum-of-Squares* (WCSS) is estimated for each cluster (WCSS is the sum of squares of the distances of each data point in all clusters to their respective centroids.) [103,104].

The fundamental idea of the elbow method is to adopt the square of the distance between the sample points in each cluster and the centroid of the cluster to give a series of K values. The Within-Cluster-Sum-of-Squares (WCSS) is adopted as a performance indicator and we can iterate over the K-value and calculate the WCSS [105]. Smaller values indicate that each cluster is more convergent. When the number of clusters is set to approach the number of real clusters, WCSS shows a rapid decline. When the number of clusters exceeds the number of real clusters, WCSS will continue to decline, but it will quickly become slower. The K value can be better determined by plotting the K-WCSS curve and by finding the inflection point where the curve yields an angle.

The elbow method is adopted as it is visual, more intuitive, and examines each data point within each cluster in comparison to the cluster’s centroid. In addition, the elbow method is mainly a decision rule, compared to other methods, such as silhouette, which is a metric used for validation while clustering. As such, it can be used in combination with the elbow method. Based on this premise, the elbow method and the silhouette method are not alternatives to each other for finding the optimal K. Rather, they are tools which can complement each other for a more confident decision [102].

**Figure 5:**
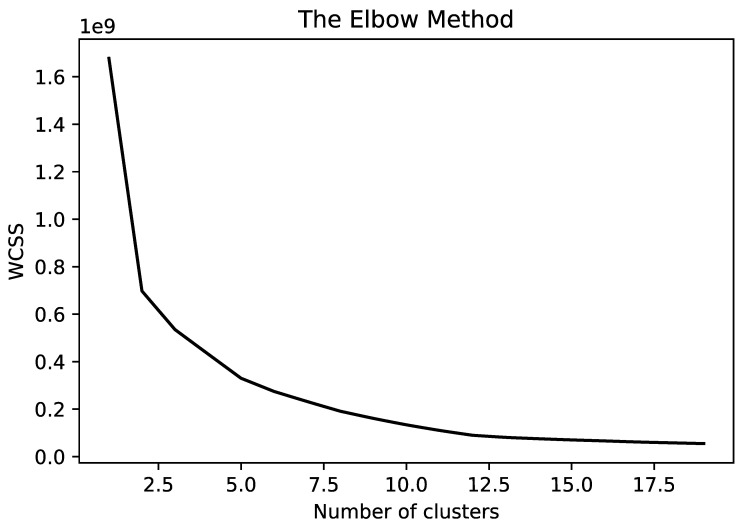
Elbow method to select number of clusters.

The corresponding graph is plotted with respect to the number of clusters and WCSS (as in Figure 5). The optimal number of clusters is the point at which increasing the number of clusters does not improve the clustering index considerably. Figure 5 demonstrates corresponding graph for all six classes or product usage activities in our dataset. Considering this figure, the optimal number of clusters selected is 6 for all classes.

### 4.4. Experiments

The unsupervised machine learning experiments were carried out based on the knowledge of the optimal number of activities (*k*) in order to gain a good understanding of the performance of unsupervised machine learning methods on each of the sensors for product use activity recognition. Given that the optimal *k* was known (as described in Section 4.3, *k* = 6), which is also equivalent to the number of classes in this case (the activity response variable), we used the “Classes to clusters evaluation” clustering mode in WEKA and applied the following three classic unsupervised machine learning algorithms (with *k* = 6, using the default options for each clustering algorithm [106]): K-means, Expectation-Maximization (EM), and Farthest First. These three unsupervised machine learning algorithms have also been explored in a prior study.

In addition, we carried out another experiment using fuzzy C-means algorithm [107,108], which was implemented in Python using skfuzzy package as they are unavailable in WEKA. We selected this additional algorithm because it enables us to consider activity as belonging to more than one discrete cluster. This means multiple concurrent activities (e.g., walking and dropping) can belong to different clusters to different degrees, given the activity is performed in real time. We anticipate that defining cluster memberships to different degrees will improve the performance of an activity-focused clustering approach. In addition, we wanted to conduct a more robust and comprehensive accuracy comparison of unsupervised machine learning algorithms with different features, complexities, and strengths. All four algorithms are further described below.

#### 4.4.1. WEKA

In WEKA, we used the Classes to clusters evaluation clustering mode when performing the clustering. Using the Classes to clusters evaluation mode, in the training phase, WEKA ignores the classes or label attribute and generates the clustering based on a selected clustering algorithm. The testing phase then proceeds after that. During the testing phase, classes are assigned to the clusters, based on the majority value of the class attribute within each cluster. Finally, the classification error is computed. Based on this assignment a corresponding confusion matrix is derived, from which we can compute the evaluation metrics (precision, recall and f-measure) described in Section 4.5.

##### K-Means

This algorithm is a well-used clustering algorithm [109]. It is a distance-based technique which functions in an iterative manner. At the initiation of the *K-means* algorithm, *k* objects would be randomly selected to be the centers of the clusters. All objects are then grouped into k clusters based on the minimum squared-error criterion (Only one assignment to one center is possible. If multiple centers have the same distance to the object, a random one would be chosen.). This helps to measure the distance between an object and the cluster center. At this point, the new mean is calculated for each cluster and then a new iteration process is carried out until the cluster centers remain the same [110,111].

Let $X=\{x_{i}\}$, i=1,2,…,n be the n objects to be clustered, $S=S_{1},S_{2},\ldots ,S_{k}$ is the set of clusters. Let μ be the mean of cluster $S_{i}$. The squared-error between $\mu_{i}$ and the objects in cluster $S_{i}$ is defined as $WCSS\left(S_{i}\right)=\Sigma_{X_{j}\in Si}{∥X_{j}-\mu_{i}∥}^{2}$. The K-means algorithm aims to minimize the sum of the squared error over all k clusters, that is $min\left(WCSS\left(S\right)\right)=argmin_{s}\sum_{i=1}^{k}\sum_{X_{j}\in Si}{∥X_{j}-\mu_{i}∥}^{2}$, where WCSS denotes the sum of the squared error in the inner-cluster. A drawback to K-means algorithm is that it requires the specification of the number of ”k“ (clusters) beforehand and also and the inappropriateness for discovering non-convex clusters [112].

##### Expectation-Maximization (EM)

This algorithm functions by determining the members of each data object according to a probability [113]. EM consists of maximization steps and expectation. The cluster probability of each object is calculated by the expectation step while the maximization step calculates the distribution parameters that maximize the likelihood of the distributions given the data [110,111]. The EM algorithm has one notable advantage, which is being stable and easy to implement. However, a drawback is that it is slow to converge and intractable expectation and maximization steps [114]. This is represented by the formulas:

$$Q\left(\theta |\theta^{\left(t\right)}\right)E_{Z}{|}_{X}{,}_{\theta }^{\left(T\right)}\left[logL\left(\theta ;X,Z\right)\right]\mathrm{and}\theta^{t+1})={\mathrm{argmax}}_{\theta }Q\left(\theta |\theta^{\left(t\right)}\right).$$

##### Farthest First

This algorithm is a fast and greedy algorithm. In summary, *k* points are initially selected as cluster centers. The first center is randomly selected. The second center is greedily selected as the point furthest from the first, while the remaining centers are obtained by greedily selecting the point farthest from the set of already chosen centers. The remaining data points are added to the cluster whose center is the closest to it [115].

For each $Xi=[x_{i},1,x_{i},2,\ldots ,x_{i}{,}_{m}]$ in D that is described by m categorical attributes, we use $f(X_{i}{,}_{j}|D)$ to denote the frequency count of attribute value $x_{i}{,}_{j}$ in the dataset. A scoring function is designed for evaluating each point, and this is defined as:

$$Score\left(X_{i}\right)=\sum_{j=1}^{m}f\left(X_{i}{,}_{j}|D\right).$$

#### 4.4.2. Fuzzy C-Means

Unlike K-means and other classical clustering algorithms in which data just belongs to a certain cluster, in fuzzy C-means clustering algorithm, data may belong to two or more clusters at certain degree [116]. The degree of membership in fuzzy C-means algorithm can accept any value from the interval [0,1]. This type of representation is more expressive of the data which includes a better detailed view of the data and relationships among clusters [117]. It may, as well, alleviate the problem associated with K-means clustering, which is basically the assignment of an appropriate cluster to data when it is on the same distance with respect to center of clusters [118], in which case, the degree of membership assigned by fuzzy C-means is equal for the two clusters.

The fuzzy C-means algorithm as proposed in 1983 is to find fuzzy membership functions which minimize the following cost function [119].

(1) $$J_{m}\left(U,R\right)=\sum_{i=1}^{N}\sum_{j=1}^{K}\mu_{ij}^{m}{\left|x_{i}-r_{j}\right|}^{2},$$

subject to:

(2) $$\mu_{ij}\in \left[0,\;1\right];\sum_{j=1}^{k}\mu_{ij}=1\;\forall i;0<\sum_{i=1}^{n}\mu_{ij}<N,\forall N,$$

where $X={\left\{x_{i}\right\}}_{i=1}^{N}$ is the set of data points, $U={\left\{\mu_{ij}\right\}}_{i,j=1}^{NK}$ is the matrix of membership degrees, k∈N is the number of clusters, $R={\left\{r_{i}\right\}}_{i=1}^{k}$ is the set of representatives, and *m* is a measure of fuzziness of the cluster. In case m→1, the fuzzy C-means becomes standard K-means method. Moreover, |.| represents the Frobenius norm of its arguments. An iterative algorithm is summarized in a few steps, as follows (Algorithm 1).


 **Algorithm 1:** Calculate fuzzy C-means.  **Require:**
$X={\left\{x_{i}\right\}}_{i=1}^{N}$ and *k*
   **return**
*U* and *R*   $U^{0}$ is randomly initialized
   **while**
$\;|U^{k+1}-U^{k}|>e$
**do**
    Calculate $r_{j}$ as follows.
                    $r_{j}=\frac{\sum_{i=1}^{n}\mu_{ij}^{m}x_{i}}{\sum_{i=1}^{n}\mu_{ij}^{m}},\;\;j=1,\ldots ,k$   
    Update $u_{ij}$ as follows.
                    $u_{ij}=\frac{1}{\sum_{i=1}^{C}{\left(\left|\frac{x_{i}-r_{j}}{x_{i}-r_{k}}\right|\right)}^{\frac{2}{m-1}}}$
   **end while**   End


Fuzzy partition coefficient (FPC) is used to find the optimal number of the clusters associated with fuzzy c-means algorithm. This algorithm measures the amount of overlap between clusters. The resulting coefficient belongs to the interval [0,1] with its maximum value at *one* corresponding to no overlap between clusters. Fuzzy partition coefficient method is a simple yet effective algorithm which computes the coefficients associated with fuzzy clustering with certain number of clusters as follows [119].

(3) $$FPC=\frac{1}{N}\sum_{i=1}^{N}\sum_{j=1}^{K}\mu_{ij}^{2}.$$

This index is computed for different number of clusters, and the number of clusters corresponding to maximum number of FPC is considered as the optimal value of clusters for performing fuzzy C-Means.

After performing clustering, each cluster is assigned to the class whose members mostly belong to. In this way, multiple clusters represent a single class. Since the distribution of data in each class may not be just around a single mean value, using multiple clusters makes it possible to find multiple separate points around which data corresponding to a class is distributed.

#### 4.4.3. Fuzzy Partition Coefficient

**Figure 6:**
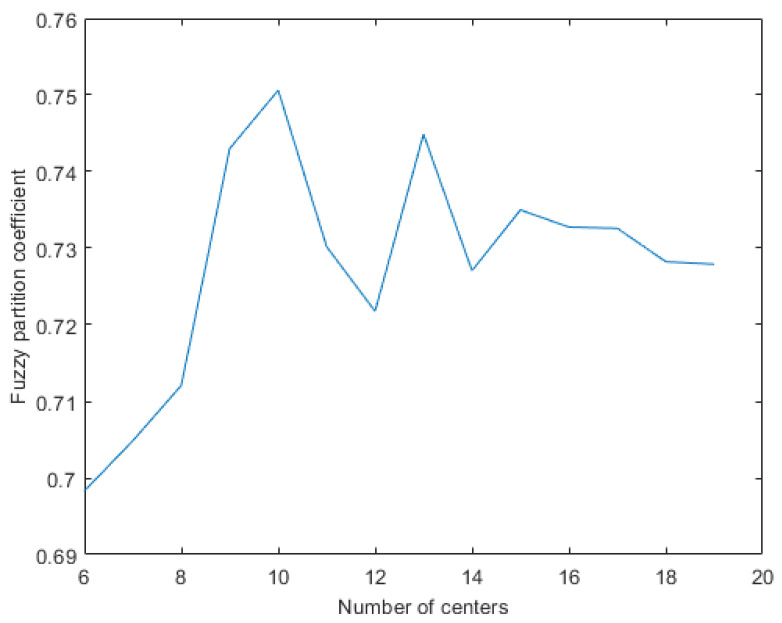
Fuzzy partition coefficient versus number of partitions.

As mentioned earlier, FPC is used for finding the optimal number of clusters for fuzzy clustering. This index is iterated versus different number of clusters. The results of this experiment are depicted in Figure 6. Higher FPC corresponds to better fuzzy C-means prediction. As can be seen from this figure, the optimal number of clusters need to be considered using fuzzy C-means is 10.

#### 4.4.4. Cluster Class Assignment

Similar to the procedure performed under WEKA for classification, the fuzzy C-means algorithm ignores class labels during training, and it builds clusters based on similarity between features, as described earlier. During the testing phase, new data points are assigned to clusters based on the fuzzy C-means learned model from the training phase—with similar data points clustered together. Predicted class labels are then assigned to each cluster, based on the majority value of the class labels associated with the data points assigned to each cluster. The corresponding confusion matrix is then derived from the real labels and the predicted ones, from which the evaluation metrics (precision, recall, and f-measure) described in Section 4.5 are measured.

### 4.5. Evaluation Metrics

Maheshwari et al. [120] used F-measure and the area under a ROC (Receiver Operating Characteristic) curve (AUC) measures for evaluating classifiers with imbalanced data set because accuracy can be misleading when used to evaluate algorithms on imbalanced data sets.

On the other hand, Shepperd et al. [121] emphasized that “*determining classification performance is more subtle than it might first appear since we need to take into account both chance aspects of a classifier (even guessing can lead to some correct classifications) and also what are termed unbalanced data sets where the distribution of classes is far from 50:50*”.

Since we are dealing with a multi-class (sitting, vibrating, walking, etc.) classification problem, we adapt the evaluation measures used by Maheshwari et al. [120] and the Matthews Correlation Coefficient (*MCC*) for dealing with imbalanced data (unequal distribution of classes) [121], taking the average measures for all classes as we cannot guarantee an even distribution of instances or occurrences per product usage activity or class in the wild.

The performance measures used in this study are described as follows:

- ***F*****-measure**: The *F*-measure performance metric uses recall and precision for performance measurement, and it is a harmonic mean between them. In practice, high *F*-measure value ensures that both recall and precision are reasonably high [120]. Precision is used to measure the exactness of the prediction set, as well as recall its completeness. These can be represented mathematically as:
  (4) $$Precision=\frac{TP}{TP+FP},$$
  (5) $$Recall=\frac{TP}{TP+FN},$$
  (6) $$F-measure=\frac{2*Precision*Recall}{Precision+Recall},$$
  where: *TP* = True Positives; *TN* = True Negatives; *FP* = False Positives; *FN* = False Negatives.
- **Matthews correlation coefficient (*MCC*)**: *MCC* is a correlation coefficient between the observed and predicted classifications. This measure takes into account *TP*, *FP*, *TN*, and *FN* values and is generally regarded as a balanced measure, which can be used even if the classes are unbalanced. This measure returns a value between −1 and +1. A coefficient of +1 represents a perfect prediction, 0 means a random prediction, and −1 indicates total disagreement between prediction and observations [121,122].
  The MCC is computed as follows:
  (7) $$MCC=\frac{\left(TP*TN)-(FP*FN\right)}{\sqrt[{}]{\left(TP+FP)(TP+FN)(TN+FP)(TN+FN\right)}.}$$

## 5. Results

Table 2 presents the empirical results from the unsupervised machine learning experiments. It shows the Precision, Recall, F-measure, and *MCC* for each of the six machine learning techniques used. The applied algorithms generated 6 clusters of similar data instances during the training phase. And in the testing phase, the algorithms attempted a 1-1 mapping of each of the actual six classes/labels shown in Table 3 to each of the generated or predicted (six) clusters. The accuracy metrics were then generated based on the correctness of this mapping.

Based on the last column in Table 2, the comparison results between the classical clustering approaches of K-means, EM and farthest first shows that only the K-means and EM clustering algorithms are able to map all 6 classes to the 6 clusters generated during the training phase. The farthest first algorithm is unable to map all classes to the 6 clusters given the high proportion of false positive and false negatives derived during the training phase of the algorithm as compared to the total number of instances in the dataset.

Furthermore, looking at the F-measure and MCC of the K-means and EM unsupervised machine learning algorithms, we can deduce that the EM algorithm (with F-measure of 79% and MCC of 0.78) outperforms the k-means algorithm (with F-measure of 57% and MCC of 0.45), both in terms of F-measure and MCC. The K-means invariably outperforms the Farthest first algorithm, given that the Farthest first algorithm was only able to correctly cluster or map 3 out of the 6 classes from the dataset to clusters, and resulted in a lower number of correctly classified instances overall (or of all 3 algorithms applied).

The results obtained for fuzzy C-means for clustering are reported with the number of clusters equal to 10 as previously obtained from FPC method in Section 4.4.3. Considering the results associated with fuzzy C-means algorithm in Table 2, it can be concluded that this approach results in superior performance when compared to the classical clustering approaches of K-means, EM and farthest first. Furthermore, the optimal number of clusters estimated in this paper for fuzzy C-means algorithm is 10, which is multiple clusters per class. This means that fuzzy C-means can effectively cluster data samples in cases where data associated with a class are distributed in multiple separate clusters.

For a deeper evaluation of the best performing algorithm, Table 3 presents the break down of the results obtained for fuzzy C-means in terms of evaluation indexes of precision, recall, f-measure, and MCC for each class, separately. It can be observed that the activities of dropping and picking up, sitting, and walking, which correspond to the classes of 1, 2, and 6, are recognized with higher performance than the other three classes of standing, stationary on side table and vibrating on surface, which correspond to classes number 3, 4, and 5. This could be because the clusters generated by fuzzy C-means for movement characteristics observed by sensors for the cases of dropping and picking up, sitting, and walking are more easily separated than the rest of classes, making them easier to be distinguished. In other words, the movement characteristics observed by sensors for two classes of standing and vibrating on a surface have more similarity to each other, making it more difficult for the software to recognize such activities, which may, in turn, also explain why the fuzzy clustering method has improved on other more static approaches.

## 6. Conclusions

Four different clustering algorithms were applied in this study for the clustering of a sensor-generated product use activity dataset. K-means algorithm is a simple and easy way to classify datasets through assumed k clusters with fixed a priori. EM algorithm is based on the probability or the likelihood of an instance to belong to a cluster following the expectation and maximization steps. The Farthest First algorithm is an iterative, fast and greedy algorithm in which the center of the first cluster is selected at random. Other than these classical clustering techniques, fuzzy C-means algorithm is also used for performing the clustering to test our assumption that results would improve if we enabled multi-activity clusters, where activities belong to multiple clusters to different degrees during real-time behavior/experiences.

The highest accuracy (in terms of both the F-measure and MCC model evaluation metrics for dealing with imbalanced datasets) obtained is 0.87 (F-measure) and 84% (MCC) for the fuzzy C-means algorithm—outperforming the other three algorithms applied on the dataset. We also show that the ability to classify different activity types is consistently high. There are no activities that the best performing model struggled to detect.

The results demonstrate that the detection of “unknown” product use activity in the wild from in-built activity sensors using unsupervised machine learning algorithms is a feasible approach. This could be applied practically to achieve the first stage of the “Chatty Factories” concept—product “experience” collection from product use in the wild, and automated methods to detect product usage activities. This allows the product design process to move beyond traditional customer feedback or manual consumer research, to include augmented information about how the product is actually used based on usage-data. This advances the manufacturing sector’s ability to redesign products such that they are better fit for purpose. Moreover, product refinement can be accelerated using the proposed methods, as designers will become less dependent on longer-term studies, such as consumer market research, from small samples of consumers. Product usage data gathered using our proposed methods can provide near real-time insights from consumers at scale—across time, space, and social cultures, enabling continuous product refinement.

## Figures and Tables

**Table 2:** Overall Precision, Recall, *F*-measure, and MCC.

	Precision	Recall	F-Measure	MCC	Classes Mapped to Clusters
K-means	0.58	0.57	0.57	0.45	All 6
Expectation-maximization (EM)	0.79	0.80	0.79	0.78	All 6
Farthest first	0.57	0.67	0.61	0.31	Only 3
Fuzzy C-means	0.87	0.87	0.87	0.84	All 6

**Table 3:** Precision, Recall, *F*-measure, and *MCC* for fuzzy C-means.

Evaluation Metrics	Class #1 Dropping and Picking Up	Class #2 Sitting	Class #3 Standing	Class #4 Stationary on Side Table	Class #5 Vibrating Surface	Class #6 Walking
Precision	0.94	0.82	0.84	0.76	0.99	0.94
Recall	1.	1.	0.69	0.99	0.53	1.
F-measure	0.97	0.90	0.76	0.86	0.69	0.97
MCC	0.96	0.88	0.72	0.84	0.69	0.96

## Data Availability

Dataset is available at: https://doi.org/10.6084/m9.figshare.11475252.v1, accessed on 3 December 2020.
